# Daiokanzoto (Da-Huang-Gan-Cao-Tang) is an effective laxative in gut microbiota associated with constipation

**DOI:** 10.1038/s41598-019-40278-2

**Published:** 2019-03-07

**Authors:** Kento Takayama, Chiho Takahara, Norihiko Tabuchi, Nobuyuki Okamura

**Affiliations:** Faculty of Pharmacy and Pharmaceutical Sciences, Fukuyama University, 1 Sanzo, Gakuen-cho, Fukuyama, Hiroshima 729-0292 Japan

## Abstract

Interindividual differences affect the purgative activities of sennoside A (SA) and Daiokanzoto (Da-Huang-Gan-Cao-Tang, DKT). In this study, we manipulated gut microbiota in mice to establish laxative responders and non-responders by feeding them a high-carbohydrate, a high-fat or a high-fibre diet. To assess the relationship between laxatives and gut microbiota, we monitored the gut microbiota before and after administering laxatives. Twenty mice per diet were divided into four groups of five mice to evaluate purgative activities of four laxative preparations, DKT, SA, SA plus rhein 8-*O*-*β*-D-glucopyranoside (SA + RG), and SA plus liquiritin (SA + LQ). Gut microbiota changes were monitored by next-generation sequencing of 16 S rRNA gene amplicons. In high-carbohydrate and high-fat diet-fed mice, DKT exerted a significantly higher purgative activity than SA alone, and RG contributed to this activity. DKT and SA + RG administration increased the Enterobacteriaceae content of gut microbiota, which was associated with an increased purgative activity. In contrast, DKT activity was significantly suppressed by high-fibre diet. Hence, diet-induced differences in gut microbiota determined the effect of DKT, which is interesting, considering that Oriental medicines are formulated for a specific functional state or “pattern”. These results demonstrated that the purgative activity of anthranoid laxatives is susceptible to diet-induced alterations in gut microbiota.

## Introduction

Constipation is a functional digestive disorder that is frequently observed in individuals worldwide. In Japan, there are more women than men with constipation, and among women, the number of young patients is already large, but the number of elderly patients dramatically higher^1^. This trend is recognized not only in Japan but also in Western countries. Cohort studies on constipation have also assessed its associations and the increased incidence of chronic kidney disease^2^ and Parkinson disease^3^, including reports on increased mortality rates^4^. Therefore, there is a medical need to improve treatment strategies for constipation is increasing.

Among available constipation medicines, stimulant laxatives are known to exert strong purgative activities. Sennosides are stimulant laxatives derived from anthraquinone that are converted by β-glucosidases in gut microbiota such as *Bifidobacterium spp*. to generate rheinanthrone, which is an active metabolite with purgative activity^5–7^. Sennoside A (SA) is the main active constituent of Daiokanzoto (Da-Huang-Gan-Cao-Tang: DKT), and its effectiveness has been confirmed in double-blinded comparative clinical trials^8,9^. DKT is a traditional Japanese medicinal formulation consisting of rhubarb and glycyrrhiza that is prescribed for “hard stool” as the constipation symptom. In our previous studies, we demonstrated that the stimulating activity of SA on gut microbiota was significantly improved when rhein 8-*O*-*β*-D-glucopyranoside (RG) from rhubarb or liquiritin (LQ) from glycyrrhiza were co-administered with SA^10–12^. Moreover, the purgative activity of SA was significantly intensified when RG and LQ were orally co-administered to mice^11,13^. These results demonstrated that the effect of these constituents on the fate of rheinanthrone generated from SA might promote the purgative activity of SA. These studies have identified beneficial interactions between the multiple constituents of DKT. However, the long-term use of anthranoid laxatives is generally thought to be possibly harmful to the gut mucosa, which leads to a condition known as melanosis coli^14^. Therefore, the Evidence-based Clinical Practice Guidelines for Chronic Constipation 2017 in Japan stated that anthraquinone laxatives should be administered for short periods^15^. Although the abnormal colonic motility induced by anthraquinone laxatives is thought to be due to injuries to the colon wall, no clear evidence relating anthraquinone laxatives to colorectal melanosis has been reported^16–18^.

Following the administration of a medicine, there are individuals who show effects (responders) and those who do not (non-responders), and this phenomenon varies between patients. Similarly, for sennosides and DKT, there are responders who experience a purgative effect and non-responders who have difficulty in purging, and interindividual differences in the purgative activity have been recognised. In Oriental medicine, DKT is thought to be adapted to treating the constipation of relatively sturdy individuals but SA, which is the main active constituent, acts via the gut microbial metabolism. Therefore, this implies that there is a close relationship between responders and non-responders to DKT and the gut microbiota in the development of purgative activity.

**Figure 1:**
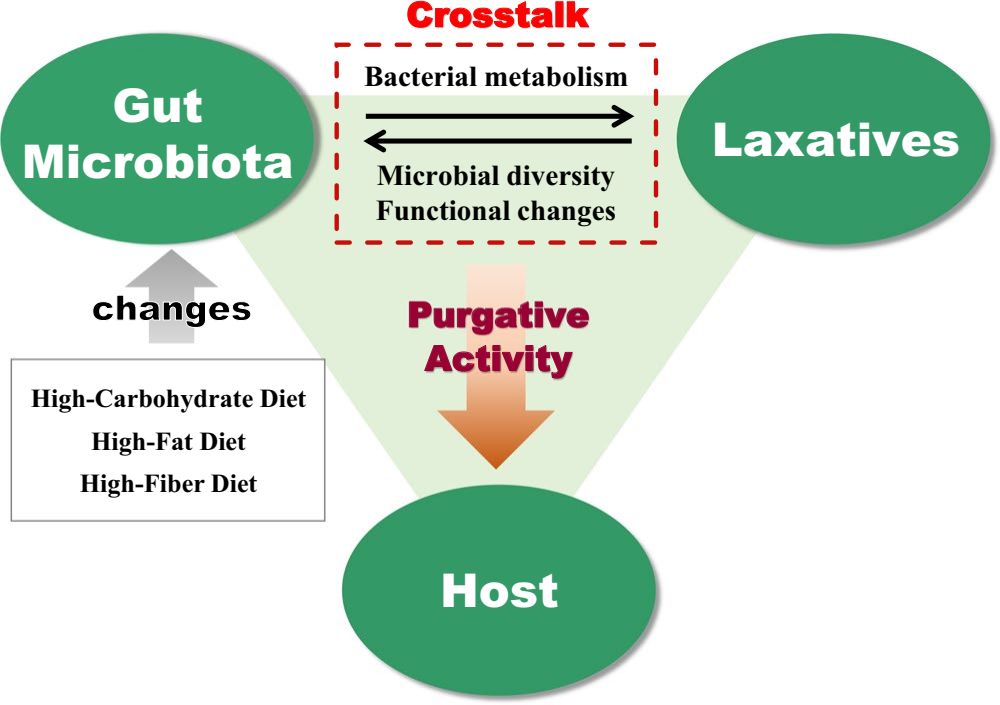
The interactions between anthranoid laxatives and the gut microbiota.

In this study, the gut microbiota was modified by feeding mice with three different diets, a high-carbohydrate (normal), a high-fat or a high-fibre diet, (Supplementary Table S1) to establish laxative responders and non-responders. Then, these controlled diet-fed groups were used to evaluate the metabolic activities of SA alone and DKT orally administered daily for 5 days, followed by the evaluation of changes in purgative activity. We assessed the change in purgative activity, as well as gut microbial diversity and function before and after laxative administration, and shed light on the crosstalk between laxative activities and gut microbiota (Fig. 1).

## Results

### Changes in Metabolic Activity of Anthranoid Laxatives Caused by Different Diets

Mice were fed three distinct diets to change the gut microbiota, and the metabolic changes of SA alone or in DKT were evaluated. The result (Supplementary Fig. S1) indicated that the metabolism of SA in DKT was significantly accelerated as compared to that of SA alone following the ingestion of any of the three feeds (*p* < 0.001). Therefore, to identify the constituent of DKT that promotes the metabolic differences, we evaluated the metabolic activity of SA in the presence of RG from rhubarb or LQ from glycyrrhiza, which accelerated the metabolic activity of SA in DKT. Consequently, the metabolism of SA was significantly accelerated by the addition of RG as compared that with SA alone (*p* < 0.001), and the effect was similar to that observed in the presence of DKT (Supplementary Fig. S1). These results indicated that the SA metabolism promoting the activity of DKT under the three dietary conditions was induced by RG.

### Changes in Purgative Activity of Anthranoid Laxatives Caused by Differences in Diets

Three groups of twenty mice each were fed three different diets for 4 weeks. Then, each diet-fed group of 20 mice was subdivided into four groups of five mice to test four laxative preparations, SA alone, DKT, SA mixed with RG (SA + RG), or SA mixed with LQ (SA + LQ), by oral administration over 5 days. The SA and DKT doses were based on our earlier report^13^. It was confirmed that these doses exerted a purgative activity that was equivalent to that observed in our previous study. In addition, RG was used at the same concentration as it is found in DKT, and LQ was used at twice the concentration as it is found in DKT to have an activity that is comparable with the RG activity. Diarrhoea was evaluated based on the purgative activity method that we typically use as described previously^11,13,19,20^. Faeces were collected and analysed at 1-h intervals for 10 h.

**Figure 2:**
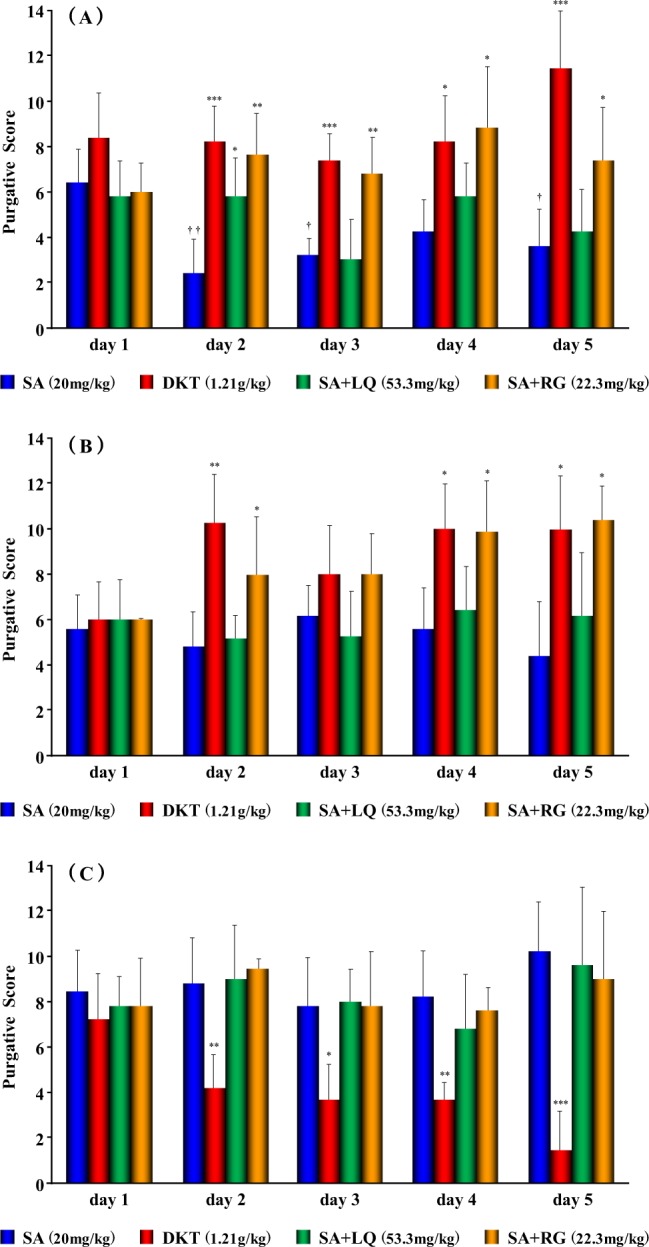
The purgative activity of anthranoid laxatives in mice fed different diets. Effects in groups fed (**A**) a high-carbohydrate, (**B**) a high-fat or (**C**) a high-fibre diet. Data points are presented as the mean ± standard deviation (SD) derived from five mice; *p < 0.05, **p < 0.01, and ***p < 0.001, a statistically significant difference in relation to sennoside A (SA) using Bonferroni’s multiple comparison test; ^†^*p* < 0.05 and ^††^*p* < 0.01, significantly different from SA on day 1 using Bonferroni’s multiple comparison test.

The purgative activity of SA in the high-carbohydrate diet-fed group was significantly suppressed (*p* < 0.05) by daily administration (Fig. 2A). On the other hand, the purgative activity of DKT was significantly promoted (*p* < 0.001) during the treatment period as compared to that on the first day of administration (Fig. 2A). Furthermore, we discovered that the purgative activity differed between the Western medicine strategy of administering SA alone and the traditional Japanese medicine practice of administering DKT. Therefore, to identify the constituent of DKT that mediated this difference, we examined the purgative activity of SA in the presence of RG and LQ. Interestingly, we demonstrated the involvement of RG in promoting the purgative activity of DKT under the high-carbohydrate diet conditions, because RG significantly promoted (*p* < 0.05) the purgative activity of SA similar to that of DKT (Fig. 2A). Furthermore, daily administration of SA alone under high-fat diet feeding did not significantly change the purgative activity (Fig. 2B). However, the purgative activity of DKT continued to be significantly promoted (*p* < 0.05) as compared with that on the first day of administration in the high-fat diet-fed group, and this activity was also found to involve RG (*p* < 0.05, Fig. 2B). Intake of the high-fibre diet maintained the purgative activity of SA at a high level throughout the 5-day treatment (Fig. 2C). In contrast, the purgative activity of DKT was significantly suppressed (*p* < 0.001) from the day after administration, and the difference was confirmed between SA and DKT (Fig. 2C). Therefore, we investigated whether LQ and RG are involved in this action, and the results showed that the purgative activity was comparable to that of SA alone (Fig. 2C). These results suggested that DKT constituents other than LQ or RG were involved in the suppression of the purgative activity of DKT in the high-fibre diet-fed mice. Generally, the gut environment is thought to be modulated by ingestion of dietary fibre, which improves bowel movements. Under such conditions, the suppression of the purgative activity of DKT has been of interest in the use of Oriental medicine. In our laboratory, we are currently investigating the inhibitory effect of the medicinal herbs constituting DKT and attempting to identify the specific active constituents that mediate this effect on the gut microbiota.

### Changes in Gut Microbiota induced by Anthranoid Laxatives

**Figure 3:**
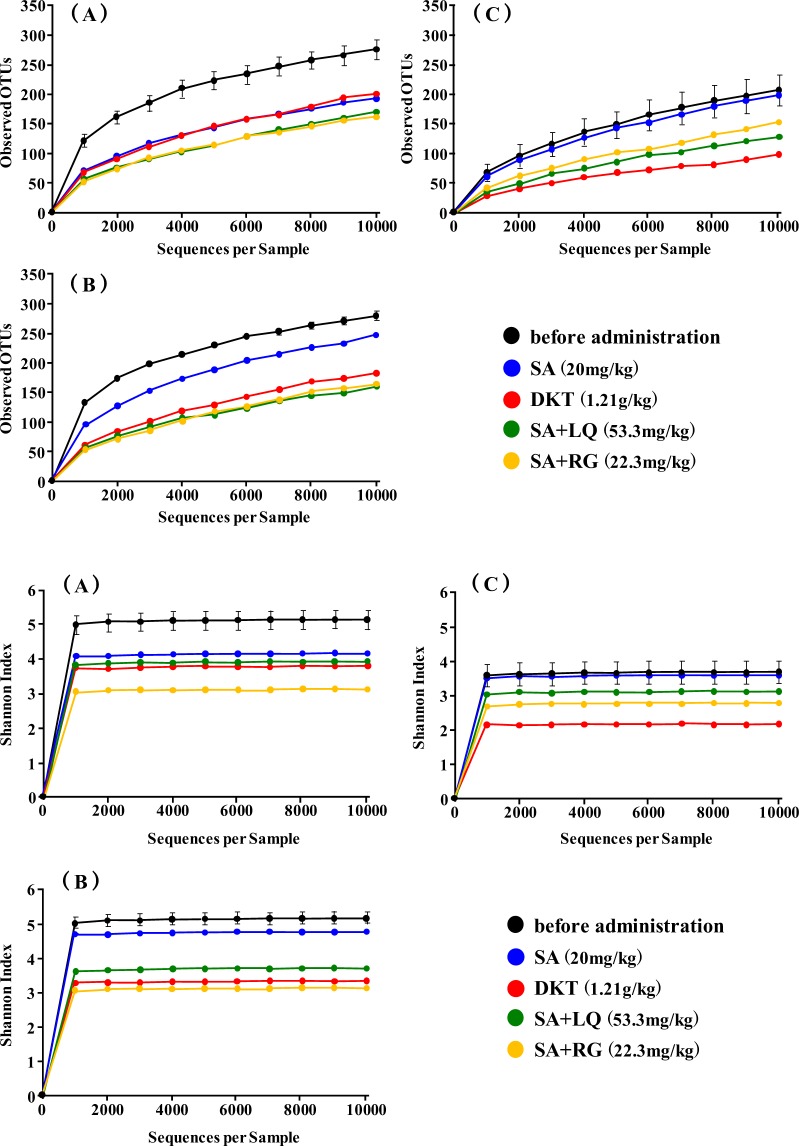
Microbial richness (operational taxonomic units, OTUs) and α-diversity of gut microbiome before and after administration of anthranoid laxatives. Effects in groups fed (**A**) a high-carbohydrate, (**B**) a high-fat or (**C**) a high-fibre diet.

To clarify whether changes in the gut microbiota due to differences in the diet and administration of laxatives were involved in the purgative activity of the laxatives, 16 S ribosomal RNA gene sequencing was performed for faecal microbiota collected from the five mice in each group using Miseq (Illumina, Seibutsu Giken Co., Ltd.) In total, 1,264,513 quality reads with an average of 52,688 reads were obtained. The observed operational taxonomic unit (OTU) changes in the α-diversity between diets or after administration of the laxatives were evaluated using the observed species (OTU richness estimation) and Shannon’s index (OTU evenness estimation). The result (Fig. 3) showed that the α-diversity decreased with the administration of laxatives for each diet except for the group fed the high-fibre diet and administered SA alone. Specifically, the α-diversity after the administration of DKT, RG, and LQ, the constituents that activate the metabolism of SA in DKT, was more reduced than after the administration of SA alone (Fig. 3).

**Figure 4:**
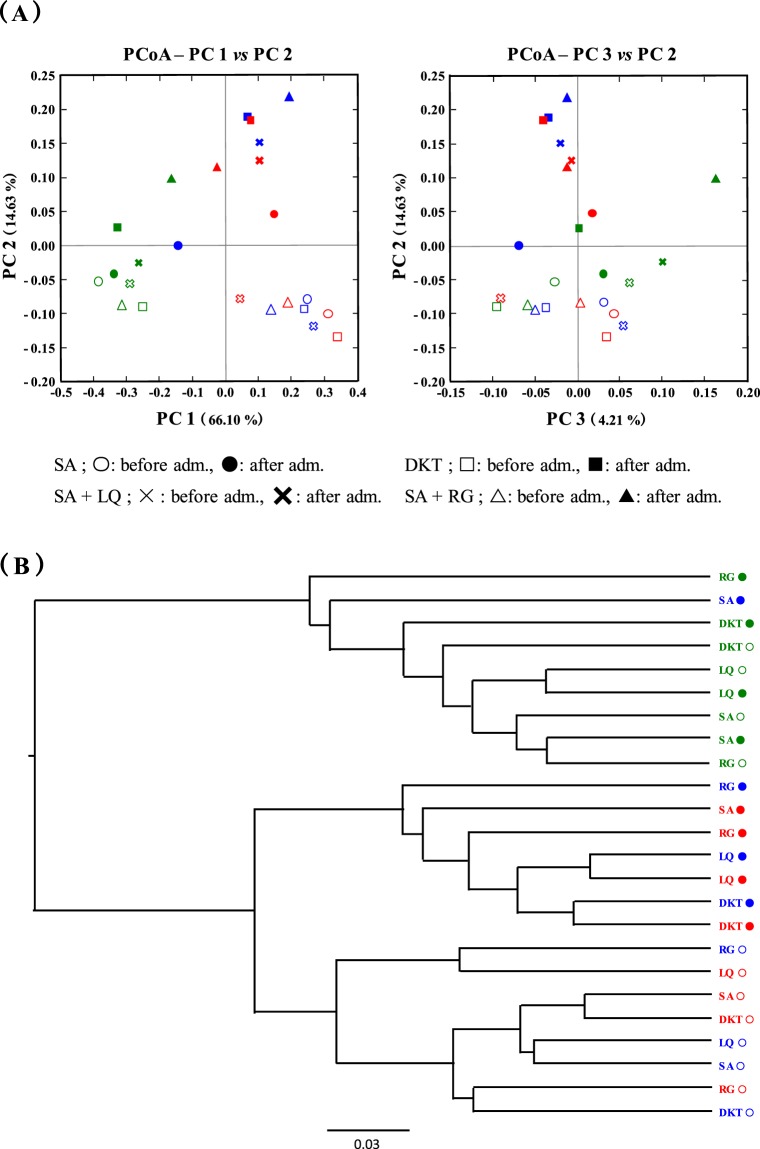
Principal coordinate analysis (PCoA) based on weighted UniFrac analysis of bacterial community structures. High-carbohydrate diet, blue; high-fat diet, red; high-fibre diet, green. Open and closed circles, before and after administration, respectively.

The overall composition of the gut microbiome was compared between each diet or before and after administration of the laxatives using β-diversity indices for the weighted UniFrac distance (Fig. 4A,B). The β-diversity before administration of laxatives indicated that the diversity changes were associated with the different diets. Especially, the β-diversities were similar under the high-carbohydrate and high-fat diet (Fig. 4A,B). When the laxatives were administered to the groups fed these two diets, the β-diversity of the gut microbiota was greatly changed as compared to that before the laxative administration (Fig. 4A,B). The β-diversity in the high-carbohydrate and high-fat diet groups after administration of DKT was assigned to the same cluster (Fig. 4B). The SA + RG group was also assigned proximate to the DKT cluster (Fig. 4B). However, the β-diversity after administration of SA alone, which exhibited a difference in the purgative activity, was assigned to different clusters for the high-carbohydrate and the high-fat diet. Especially, the β-diversity of the gut microbiota after administration of SA alone in the high-carbohydrate diet-fed group was similar to that of the high-fibre diet-fed group, which suppressed the purgative activity of DKT. In contrast, the β-diversity of the gut microbiota after administration of the laxatives to the high-fibre diet-fed group was not significantly changed from before the laxative administration (Fig. 4A,B).

**Figure 5:**
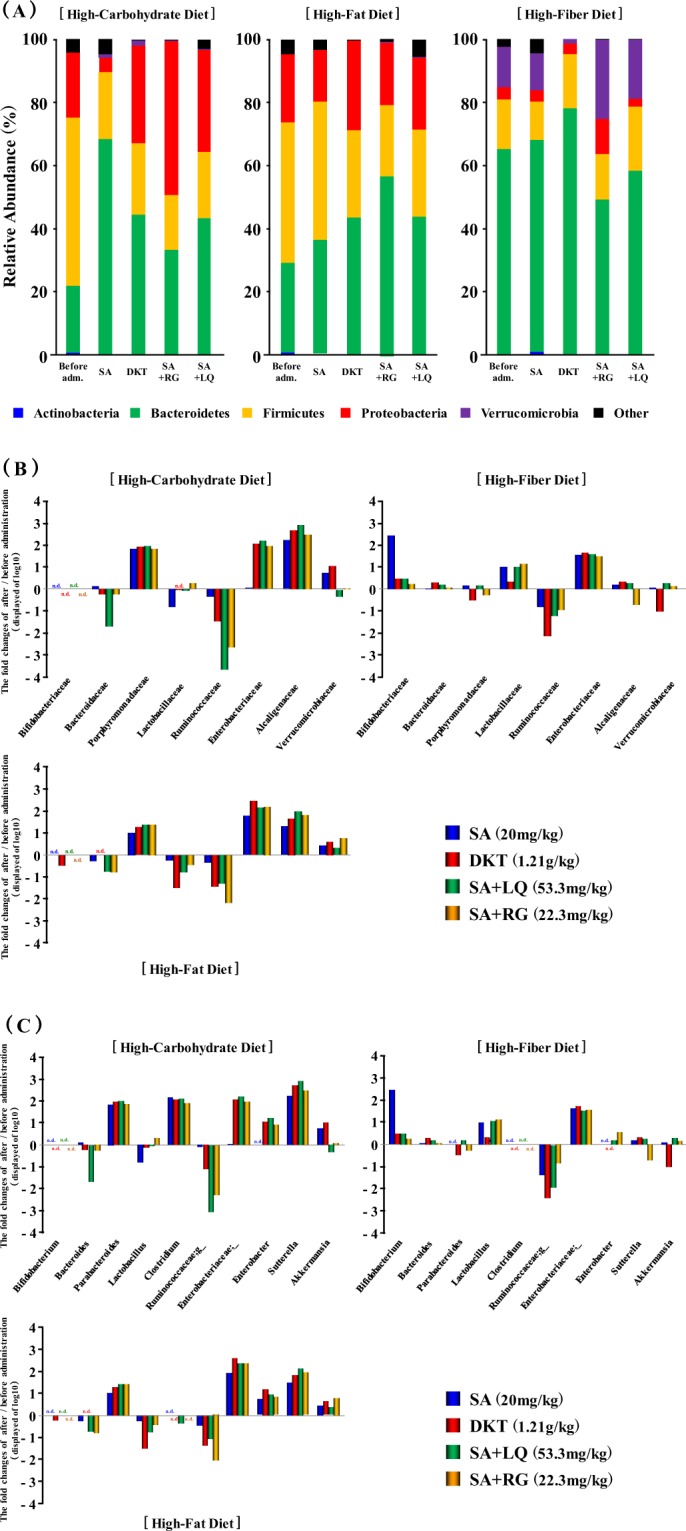
Gut microbial abundance before and after administration of anthranoid laxatives. Effects in groups fed (**A**) a high-carbohydrate, (**B**) a high-fat or (**C**) a high-fibre diet.

The differences in the gut microbial composition were taxonomically evaluated at the phylum level. The abundance of the phylum Bacteroidetes increased significantly after laxative administration as compared to that before administration regardless of the laxative tested in the high-carbohydrate and high-fat diet-fed groups (Fig. 5A). In contrast, the phylum Firmicutes barely decreased after administration of SA alone with the high-fat diet (Fig. 5A). In the high-fibre diet group, the phylum Verrucomicrobia was found to have greatly decreased, especially after administration of DKT, which suppressed the purgative activity (Fig. 5A). These results demonstrated that the gut microbiota was dynamically changed by the administration of anthranoid laxatives.

The taxonomic changes in the microbial community were also evaluated at the family and genus levels. Regardless of the laxative preparation, the family Porphyromonadaceae was shown to have increased by 100- and 10-fold in the groups fed the high-carbohydrate and high-fat diets, respectively (Fig. 5B). This was thought to be associated with an increase in the genus *Parabacteroides* (Fig. 5C). The family Alcaligenaceae increased about 100-fold with both diets, and the genus *Sutterella* also increased similarly (Fig. 5B,C). Intake of the high-fibre diet did not significantly change the levels of these bacteria. In contrast, the family Ruminococcaceae was found to have decreased by the administration of laxatives in all three diets (Fig. 5B). Interestingly, after the administration of SA alone, the purgative activity was suppressed in the high-carbohydrate diet-fed group, whereas the family Enterobacteriaceae showed little change but with DKT, which maintained or promoted the purgative activity, it was increased close to 100-fold (Figs 2 and 5B). The family Enterobacteriaceae increased almost 100-fold in all administration groups including those fed the high-fat and high-fibre diets. The genus *Bifidobacterium*, which is considered to be involved in the SA metabolism, was increased by the administration of laxatives in the high-fibre diet-fed group and increased by nearly 100-fold after the administration of SA alone (Fig. 5C). In addition, the family Verrucomicrobiaceae was nearly 10-fold reduced after DKT administration in the high-fibre diet-fed group, and the genus *Akkermansia* was also reduced. These results suggested an association between the suppression of the purgative activity of DKT and the decrease in the genus *Akkermansia*. All sequence data were deposited at the DDBJ Sequence Read Archive (https://www.ddbj.nig.ac.jp/dra/index-e.html) under accession number (DRA008020).

### Functional Changes in Gut Microbiota Induced by Anthranoid Laxatives

**Figure 6:**
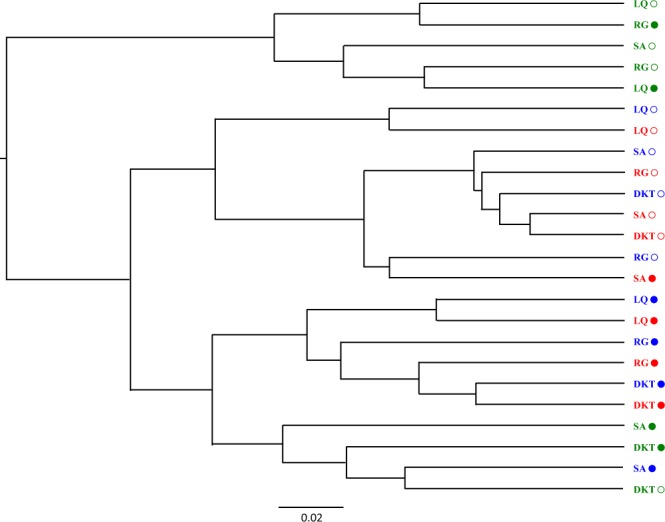
The functional changes in gut microbiota induced by differences in diets, administration of anthranoid laxatives, or both identified using PICRUSt. High-carbohydrate diet, blue; high-fat diet, red; high-fibre diet, green. Open and closed circles, before and after administration, respectively.

Potential differences in the function of the gut microbiota were examined using the Phylogenetic Investigation of Communities by Reconstruction of Unobserved States (PICRUSt) software^21^. To measure the functional similarity of microbial communities, we used the Bray-Curtis index, which is used to evaluate the difference in properties among actual crowds. The result (Fig. 6) confirmed that the administration of DKT, SA + RG, and SA + LQ to the high-carbohydrate and high-fat diet-fed groups changed the diversity of the gut microbiota. Furthermore, the functional diversities were also assigned to clusters that differed from those observed before the administration of laxatives (Fig. 6). Similar to the change in gut microbiota, even in the high fibre diet-fed groups, no significant change in function was observed before and after the administration of the three types of laxative. In addition, the functional change after administration of SA alone in the high-carbohydrate diet-fed group was assigned to different clusters similar to those of the diversity of gut microbiota change (Figs 4 and 6). However, in the high-fat diet-fed group, the diversity of function was assigned to a cluster closer to that before administration, despite the change in the diversity of gut microbiota (Figs 4 and 6). Conversely, in the high-fibre diet-fed group, the diversity of function after administration of SA alone was classified as a distant cluster although the diversity of the gut microbiota was maintained following administration. This observation suggested that the change in gut microbiota, including its functions may have affected the maintenance of the purgative activity of SA alone.

## Discussion

In this study, we determined the effect of differences in the gut microbiota on the purgative activity of DKT or SA using a mouse model established by feeding mice various diets, which differentially changed the gut microbiota. Furthermore, laxatives were administered for 5 days and the changes in the purgative activity were monitored. The results demonstrated, for the first time, that the change in gut microbiota was caused by differences in the diet and, therefore, the purgative activity of DKT and SA differed.

Zhu *et al*.^22^ reported that the α-diversity of the gut microbiota tended to increase in patients with constipation compared to those without constipation. Furthermore, the ecological diversities of the faecal microbiome of patients who were constipated differed from those who were not. In addition, the phylum Bacteroidetes was significantly decreased in patients with constipation patients compared to those who were not constipated, and the family Ruminococcaceae was significantly increased in patients with constipation^22^. Vandeputte *et al*.^23^ has shown that harder stool is associated with higher α-diversity, and the *Ruminococcus* and *Bacteroides* enterotypes were significantly higher. Based on these reports, the results of this study showed that the OTUs in the gut microbiome of the groups fed high-carbohydrate and high-fat diets before the administration of laxatives were higher than that of the high-fibre fed group (Fig. 3). In our study, in the groups fed high-carbohydrate and high-fat diets, the phylum Bacteroidetes showed lower abundance levels than the phylum Firmicutes (Fig. 5A). Therefore, we thought that this was similar to the gut microbiota of patients with constipation.

After establishing the different gut microbiota using the different diets, we investigated the changes in purgative activity and gut microbiota induced by administering DKT and SA. The purgative activity of daily administration of DKT was significantly promoted in the high-carbohydrate diet-fed group, which is thought to be similar to the gut microbiota of patients with constipation. Moreover, the purgative activity of SA alone was significantly suppressed (Fig. 2A). The gut microbiota after administration of each laxative to the high-carbohydrate diet-fed group showed an increase and decrease in the phyla Bacteroidetes and Firmicutes, respectively (Fig. 5A). The family Ruminococcaceae, which is thought to be abundance in hard stools, was not significantly changed by administration of SA alone. In contrast, it was greatly reduced by nearly 100-fold following the administration of DKT (Fig. 5B). Therefore, it has been demonstrated that the laxative effect of DKT, which is prescribed for constipation associated with symptoms of “hard stool” in Oriental medicine, was associated with the change in gut microbiota. Interestingly, the family Enterobacteriaceae was increased by nearly 100-fold following the administration of DKT to the group fed the high-carbohydrate diet, as opposed to the almost no change observed after administration of SA alone (Fig. 5B). Similarly, the purgative activity of SA alone was maintained in the group fed the high-fat diet, which is considered to be similar to the gut microbiota of patients with constipation (Fig. 2B). Moreover, the increase in the family Enterobacteriaceae was similar to that observed with the administration of DKT. The genus *Enterobacter* and unclassified Enterobacteriaceae were increased (Fig. 5C), which strongly suggested that the abundance of the family Enterobacteriaceae induced by the administration of laxatives influenced the purgative activity. The family Enterobacteriaceae includes diarrheal pathogens such as *Shigella* and *Salmonella*. Furthermore, *Citrobacter rodentium*, a close relative of the human diarrheal pathogen, enterohemorrhaic and enteropathogenic *Escherichia coli*, has been demonstrated to cause colonic hyperplasia and inflammation^24,25^. These diarrheal pathogens were not detected in this study, and we assumed that the genera of the family Enterobacteriaceae, which were unclassified, might include similar bacteria. In conclusion, this study revealed an association between the abundance of the family Enterobacteriaceae and the purgative activity.

We investigated the cause of differences in the purgative activity of DKT and SA alone in the high-carbohydrate and high-fat diet-fed groups and confirmed the influence of RG and LQ on the purgative activity of SA and the change in gut microbiota. The RG content of rhubarb has been assumed to intensify the activation of SA by accelerating its synthesis and the activity of the SA metabolic enzyme derived from *Bifidobacterium*^11,12^. LQ is the flavonoid component of glycyrrhiza that has been reported to intensify the activation of SA by accelerating the activity of the SA metabolic enzyme, similar to RG^10,13^. As a result, the purgative activity of SA + RG was significantly accelerated from day 2 in the high-carbohydrate and high-fat diet groups, similar to the purgative activity of DKT (Fig. 2A,B). In addition, the change in the gut microbiota after administration of SA + RG was revealed to be identical to that observed with DKT that greatly increased the family Enterobacteriaceae and changed the functional diversity (Figs 5B and 6). In conclusion, it has been assumed that the enhancement by DKT in the high-carbohydrate and high-fat diet groups is mediated by the effects of RG on the family Enterobacteriaceae and the accompanying change of diversity of function. In previous studies, it has been demonstrated that the action of RG is attributable to the anthraquinone core structure, which is the aglycone part of RG^11^. DKT contains several anthraquinone constituents other than RG, such as rhein, emodin, and aloe-emodin^11^. Therefore, it was strongly suggested that these constituents caused the difference in the purgative activity of DKT and SA alone. Although LQ did not influence the purgative activity of SA, it affected the gut microbiota similar to DKT (Figs 3–5). Therefore, the flavonoid was suggested to affect the alteration of the gut microbiota.

Although the purgative activity of SA alone was maintained in the high-fibre diet group, that of DKT was significantly suppressed (Fig. 2C). RG and LQ were not involved in this suppressive action and, therefore, it was suggested that DKT constituents other than RG and LQ were involved. Although the genus *Akkermansia* was decreased by administration of DKT to the high-fibre diet group (Fig. 5C), no significant difference was observed in the change of β-diversity of the gut microbiota and function (Figs 4 and 6). Presently, the effect of suppressing the purgative activity of DKT in the high-fibre diet group is under investigation to determine the specific constituent mediating this effect and the potential involvement of the gut microbiota changes.

Constipation has been neglected despite its considerable impact on the quality of life (QOL) because it has not been considered a life-threatening disease. Recently, however, constipation has been shown to increase the risk of progression of chronic kidney disease (CKD) and end-stage renal failure (ESRD)^2^. Mishima *et al*. have suggested that lubiprostone, commonly used for the treatment of constipation, ameliorates the progression of CKD and the accumulation of uremic toxins by improving the gut microbiota and intestinal environment^26^. Parkinson disease is known to possibly affect the autonomic nervous system, which may cause constipation^27–29^. Savica R *et al*.^27^ suggested that the occurrence of constipation as early as ≥20 years before the onset of motor symptoms is associated with an increased risk of Parkinson disease. Therefore, it is considered that constipation could be one of the earliest markers of the onset of Parkinson disease development^28,29^. Sampson *et al*.^30^ revealed that the gut microbiota regulated movement disorders in mice and alterations in the human microbiome represent a risk factor for Parkinson disease^30^. Pulikkan J *et al*.^31^ have suggested that children with ASD were often constipation compared to healthy children. The gut microbiota of children with ASD showed a change in it similar to other references for patients with constipation who the phylum Bacteroidetes decreased^31^. In conclusion, the relationship between constipation and various diseases has been clarified, and the importance of treating constipation is becoming increasingly evident.

Saline, osmotic, and other laxatives such as lubiprostone, which activates intestinal luminal Cl^−^ secretion and water motility, are also widely used. In this study, SA, which is also a representative stimulant laxative, and DKT, which is an herbal medicine containing SA as the main active constituent, were used to study the crosstalk between diet-induced gut microbiota and purgative activity. In modern medicine, SA is prescribed according to Western medicine diagnostic criteria, but DKT is prescribed according to Oriental medicine diagnostic criteria. In the present study, under the gut microbiota conditions similar to those in individuals with constipation, the effect of DKT was promoted by RG. In contrast, under gut microbiota conditions requiring no laxatives such as the high-fibre diet group, the purgative activity of SA alone was maintained, but that of DKT was suppressed. Taken together, these results suggest that an important finding of this study is that the diet-induced differences in gut microbiota determined the symptoms, and a “pattern” was revealed, which was an indication for traditional Japanese medicine. Although the relevance of the pattern and gut microbiota has been considered previously, this study was the first to present detailed data suggesting a scientific basis. In Oriental medicine, “healing” is not just achieved by the medical treatment such as herbal medicines, acupuncture, and moxibustion, but it is thought that everyday habits such as regular diet and defecation, are crucial to achieving a “cure”. In addition, Rothschild *et al*.^32^ also demonstrated that the gut microbiota composition is hardly related to the interindividual genetic differences of the host, but it reflects the lifestyle such as diets, drugs, or both^32^. It can be inferred from this study that the difference in the composition of gut microbiota due to lifestyle habits greatly affected the formation of the pattern. This study also highlights the fact that the purgative activity of anthranoid laxatives is susceptible to alteration by diet-induced gut microbiota. Recent evidence indicates that the anti-PD-1 efficacy is controlled by gut microbiota in basic and clinical studies^33–35^. Considering these reports, we propose that the interaction between the gut microbiota and numerous medicines is as critical as that between the gut microbiota and the traditional Japanese medicine evaluated in this study.

## Materials and Methods

### Materials

The herbal medicine formulation materials for the decoction in DKT, rhubarb (kinmon-daio in Japanese, Lot No. 100901) and glycyrrhiza (touhoku-kanzo in Japanese, Lot No. 050202) were purchased from Tochimototenkaido (Osaka, Japan). SA was purchased from Wako Pure Chemical Industries (Osaka, Japan). RG was isolated from rhubarb and was identified according to the method of a previous study^11^. Liquiritin was isolated from glycyrrhiza, and its structure was identified by comparing its ^1^H and ^13^C NMR data with those reported in the literature^36^. Ultrapure distilled water was prepared with deionized-distilled water. All other reagents and chemicals were analytical grade commercial products.

### Animal Preparation

Sixty male C57BL/6 N mice, weighing 30–40 g, were obtained from SHIMIZU Laboratory Supplies (Kyoto, Japan) and housed under a 12-h light/dark cycle at 21 to 24 °C for at least 1 week before the experiments. Mice were given *ad libitum* access to food and water before the experiments. After an acclimation period of 1 week, the 60 mice were divided into three groups of 20 mice each, and they were fed for 4 weeks on three different diets (Supplementary Table S1): high-carbohydrate (normal), high-fat or high-fibre diets. The three diets were purchased from Research Diets, Inc. (New Brunswick, NJ, USA). Then, these mice were used in the subsequent experiments. The animal studies were approved by the Research Ethics Committee of the Fukuyama University.

### Assessment of Metabolic Activity of Anthranoid Laxatives induced by Mouse Gut Microbiota

Fresh faeces collected from mice were homogenized in 20 volumes of 0.01 M potassium phosphate buffer (pH 7.4) by bubbling with CO_2_ gas to eliminate the air, and the sediments were removed by filtration through gauze. The faecal suspensions were used as the gut microbiota mixtures. DKT (20 mg/mL) was prepared with 0.01 M potassium phosphate buffer and 0.5% sodium hydrogen carbonate. SA (0.4 mM) was prepared with 0.01 M potassium phosphate buffer. RG and LQ, each at a concentration of 2 mM, were prepared with 0.5% sodium hydrogen carbonate. The assay samples were prepared by mixing equal amounts of SA and each constituent sample. Tubes containing the assay samples (0.25 mL) and the faecal suspension (1 mL) were incubated at 37 °C for 24 h under anaerobic conditions. Anaerobic procedures were carried out using an anaerobic jar with an AnaeroPack (Mitsubishi Gas Chemical, Tokyo, Japan). The reaction was immediately stopped by adding 0.425% v/v phosphoric acid in methanol. After centrifugation at 1500 *g* for 5 min, the supernatant was passed through Minisart RC 15 (Sartorius, Goettingen, Germany) and subjected to high-performance liquid chromatography (HPLC). The metabolic rate was calculated as the percentage of the content of SA in the incubation mixture compared with that in the blank. The HPLC analysis was carried out using the same method in our previous study^20^.

### Assessment of Purgative Action of Anthranoid Laxatives

Mice were isolated in a wire-bottomed cage covered with a beaker (11 × 15 cm), which was placed on blotting paper. The faeces were collected 1 h before the daily administration of each laxative, and only the mice that excreted normal faeces were used. SA (40 mg/kg) was prepared in 0.01 M potassium phosphate buffer. RG and LQ were prepared at doses of 44.6 and 106.6 mg/kg, respectively, in 0.5% sodium hydrogen carbonate. SA + RG and SA + LQ were mixed at equal amounts and orally administered to mice in a single dose. DKT (1.21 g/kg) was prepared with 0.01 M potassium phosphate buffer and 0.5% sodium hydrogen carbonate, and orally administered to mice in a single dose. After oral administration of the samples, the faeces were collected at 1-h intervals for 10 h. The faeces in the worst condition were graded for their consistency levels as follows: 0, normal; 1, soft; and 2, unformed^11,13,19,20^. The faeces score was the mean value of the total consistency level of every hour in each mouse. This experiment was conducted over 5 days.

### Sample Collection, DNA Extraction, and Quantitative Analysis of Gut Microbiome Composition

Faecal samples (200 mg in each mouse) were mechanically lysed using a Cell Destroyer PS2000 (Bio Medical Science Co., Ltd., Tokyo, Japan). Briefly, total DNA from the lysate was extracted using phenol/chloroform/isoamyl alcohol, precipitated, and washed with ethanol. Then, the DNA pellets were resuspended in Tris-EDTA buffer with RNase A (Sigma-Aldrich, St. Louis, MO). Furthermore, the DNA samples were purified using the High Pure PCR Template Preparation kit (Roche Diagnostics, Mannheim, Germany) and suspended in 200 μL elution buffer according to the manufacturer’s instructions. The library preparation and 16 S ribosomal RNA gene sequencing were outsourced to Seibutsu Giken Co., Ltd (Kanagawa, Japan). Briefly, the V1–V2 region of the bacterial 16 S rRNA gene was amplified using PCR with a bacterial universal primer set (27F-mod: 5′-AGRGTTTGATYMTGGCTCAG-3′ and 338 R: 5′-TGCTGCCTCCCGTAGGAGT-3′). The 16 S rRNA genes were subjected to paired-end sequencing using Miseq sequencer (Illumina, California, USA). The paired-end reads with Q scores ≥20 were joined using the software package QIIME v 1.9.1. The UCHIME algorithm was used to cluster the filtered sequences into OTUs based on a ≥97% similarity threshold. The sequences were checked for chimeras using UCHIME. Representative sequences from each OTU were taxonomically classified using the Greengene taxonomic database v 13_8. OTUs were used for α-diversity estimation of the observed species and Shannon diversity. PCoA plots were calculated using the OTUs from each sample based on weighted UniFrac distances. The functional changes in gut microbiota were determined using the PICRUSt software and indicated as Bray-Curtis distances.

### Statistical Analysis

Data are presented as the means ± standard deviation (SD). Statistical comparisons of more than two groups were performed using the Dunnett’s test or Bonferroni’s multiple comparison test in GraphPad Prism 5.0 (GraphPad Software Inc.). A *p* < 0.05 was considered statistically significant.

### Ethical Approval

The research reported in this study involved mice. All experimental protocols were approved by the Research Ethics Committee of the Fukuyama University and conducted in accordance with the Guidelines for Animal Experimentation of the Fukuyama University (H27-animal-13).

## Supplementary information

Supplementary Figure S1

Supplementary Table S1
