# Supplementary material for: Daiokanzoto (Da-Huang-Gan-Cao-Tang) is an effective laxative in gut microbiota associated with constipation
Source: Sci Rep. 2019 Mar 7;9:3833. doi: 10.1038/s41598-019-40278-2 (PMC6405880; doi:10.1038/s41598-019-40278-2)
Supplement: Supplementary file 1 — Supplementary Figure S1 [file 41598_2019_40278_MOESM1_ESM.pdf]

Daiokanzoto (Da-Huang-Gan-Cao-Tang) is an effective laxative in gut microbiota associated with constipation

Kento Takayama*, Chiho Takahara, Norihiko Tabuchi, and Nobuyuki Okamura

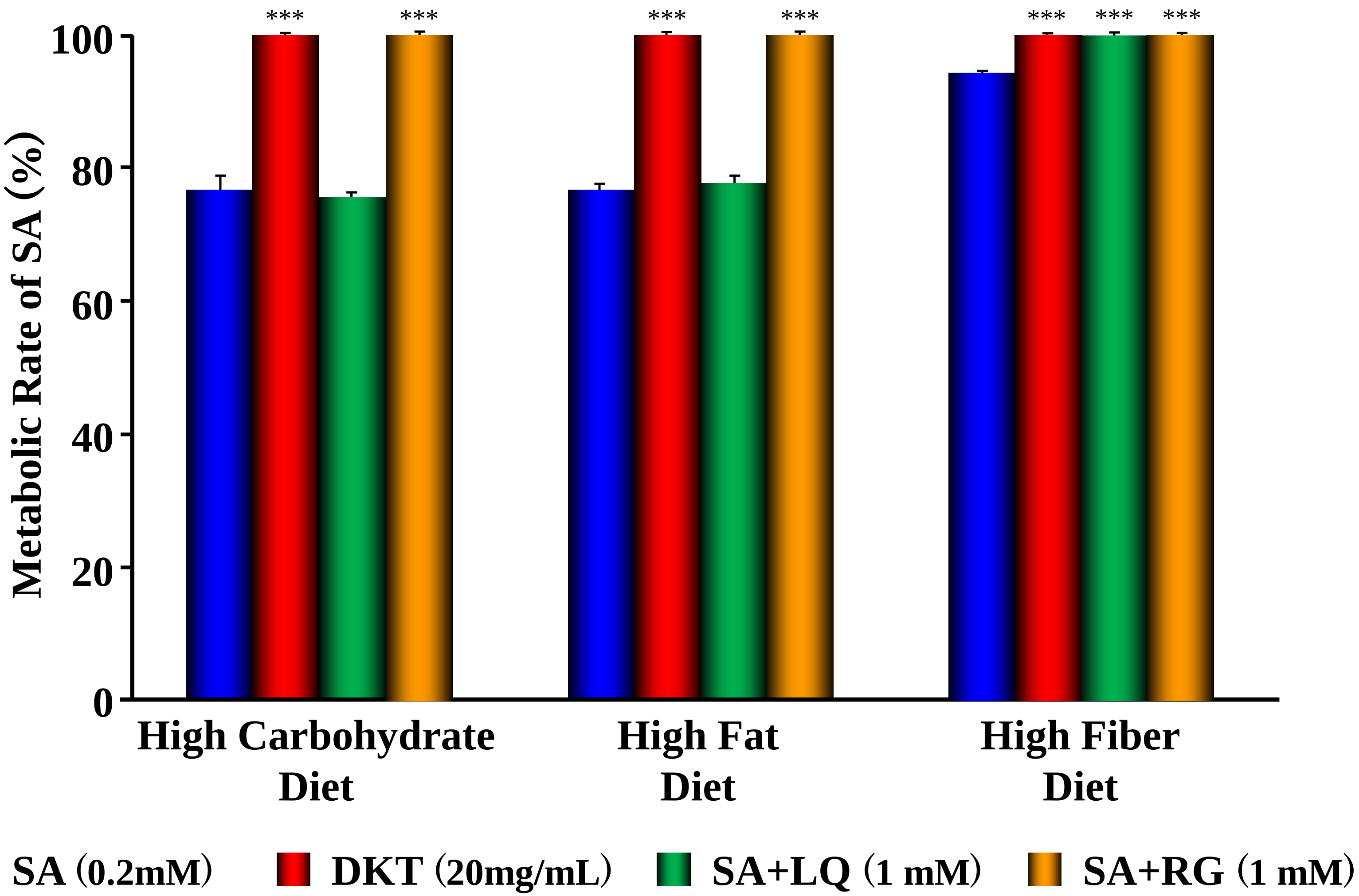

Supplementary Figure S1. Metabolic activity of anthranoid laxatives.

Values are presented as the mean ± standard deviation (SD) of three samples. ****p* < 0.001, a statistically significant difference in relation to sennoside A (SA) using Dunnett’s test.
