# Supplementary material for: Daiokanzoto (Da-Huang-Gan-Cao-Tang) is an effective laxative in gut microbiota associated with constipation
Source: Sci Rep. 2019 Mar 7;9:3833. doi: 10.1038/s41598-019-40278-2 (PMC6405880; doi:10.1038/s41598-019-40278-2)
Supplement: Supplementary file 2 — Supplementary Table S1 [file 41598_2019_40278_MOESM2_ESM.pdf]

Daiokanzoto (Da-Huang-Gan-Cao-Tang) is an effective laxative in gut microbiota associated with constipation

Kento Takayama*, Chiho Takahara, Norihiko Tabuchi, and Nobuyuki Okamura

Supplementary Table S1. Composition of diets

Product	High-Carbohydrate (Normal Diet, D12450H)		High-Fat (D12451)		High-Fiber (D14053101)	
	gm%	kcal%	gm%	kcal%	gm%	kcal%
**Carbohydrate**						
Corn Starch	452.2	1809	72.8	291	452.2	1809
Maltodextrin 10	75	300	100	400	75	300
Sucrose	172.8	691	172.8	691	172.8	691
**Fat**						
Soybean Oil	25	225	25	225	25	225
Lard	20	180	177.5	1598	20	180
**Fiber**						
Cellulose, BW200	50	0	50	0	50	0
Inulin	0	0	0	0	117	117
